# Successful medical management of a pituitary macroadenoma with features of resistant acromegaly and hyperprolactinemia using pasireotide

**DOI:** 10.5339/qmj.2024.17

**Published:** 2024-03-14

**Authors:** Khaled Ahmed Baagar, Amna Sadiq, Adeel Ahmad Khan, Zeinab Dabbous, Zaina Rohani

**Affiliations:** 1 Diabetes and Endocrine Department, Hamad Medical Corporation, Doha, Qatar Email: kbaagar@hamad.qa; 2 Radiology Department, Hamad Medical Corporation, Doha, Qatar

**Keywords:** pituitary macroadenoma, hyperprolactinemia, acromegaly, pasireotide, case report

## Abstract

Background: The somatostatin analog, pasireotide, is used for the treatment of acromegaly after the failure of surgery and/or first-line medical treatment.

Case Presentation: A 48-year-old male reported that during a workup for obesity in his home country, hyperprolactinemia was diagnosed and a 3.5 × 3.5 cm pituitary macroadenoma was identified on pituitary MRI. He received cabergoline for 6 months; then he was lost to follow-up. He presented at our Endocrine clinic 2 years later for treatment of obesity (BMI 49.5 kg/m^2^). Biochemical workup revealed that in addition to hyperprolactinemia (7,237 [normal: 85–323 mIU/L), he had acromegaly, evident by elevated insulin-like growth factor 1 (IGF-1) level (450 [normal: 88–210 µg/L]), and a positive growth hormone suppression test, secondary hypothyroidism, and secondary hypogonadism. Pituitary MRI showed that the adenoma encased parts of the left and right internal carotid arteries and encroached on the optic chiasm. Surgical excision was therefore not feasible. He was treated with cabergoline and later, long-acting release (LAR) octreotide. Prolactin levels were reduced with cabergoline, but IGF-1 levels did not respond to octreotide, and it was discontinued. The patient abandoned radiotherapy after two sessions. He was started on LAR pasireotide 40 mg every 4 weeks and continued on cabergoline 0.5 mg per week. His biochemical response was dramatic, with a near normalization of IGF-1 levels in 3 months. After 6 months from starting pasireotide, we increased cabergoline dose from 0.5 mg/week to 3 mg/week. Three months later, IGF-1 level was normalized. The patient developed type 2 diabetes as a side effect of pasireotide; however, this was well-controlled with medications.

Conclusions: The case suggests that pasireotide can provide marked biochemical improvement in acromegaly after the failure of both cabergoline monotherapy and cabergoline plus octreotide. This further confirms a potentially efficacious treatment regimen in treatment-resistant acromegaly with hyperprolactinemia.

## Introduction

Pituitary adenomas are categorized based on the characteristic cell type from which the adenoma originates. These include lactotroph, somatotroph, corticotroph, gonadotroph, thyrotroph, and null-cell (non-secretory) adenomas.^1^ Prolactinomas are the most frequently encountered pituitary tumor, accounting for around 50% of pituitary tumors.^2−4^ Prolactinomas are benign adenomas resulting in an overproduction of prolactin.^1^ When the tumor is large — a diameter exceeding 1 cm is classified as a macroadenoma — the presenting symptoms are often visual field defects and headaches. The primary treatment goal for most patients with prolactinomas is to restore normal prolactin secretion and reduce tumor size. The usual therapeutic options include observation, medical therapy with dopamine agonists, transsphenoidal or transcranial surgery, and radiotherapy.^1^ Dopamine agonists, especially cabergoline, are generally highly effective in normalizing prolactin levels; however, treatment resistance can occur, with a subset of patients having stubbornly elevated prolactin levels.^1^ Potential subsequent-line treatments for prolactinomas include the somatostatin analogs octreotide and pasireotide.^1,5−8^

Acromegaly results from excessive growth hormone (GH) secretion from pituitary tumors.^1^ High levels of circulating GH and insulin-like growth factor 1 (IGF-1) are caused mostly by GH-secreting pituitary adenomas or “somatotroph adenomas”.^1^ Somatotroph adenomas can also cause hyperprolactinemia by secreting prolactin or by compressing the pituitary stalk, as well as hypopituitarism in at least one axis by damaging the pituitary gland.^4,9^ Mixed somatotroph-lactotroph adenomas can directly secrete prolactin, contributing to hyperprolactinemia.^9,10^ A recent cohort study found that hyperprolactinemia and hypopituitarism were common in acromegaly, occurring in 39% and 35% of patients, respectively.^9^ Transsphenoidal surgery (TSS) is usually the first-line treatment for most patients. Somatostatin analogs are used as second-line treatments to lower GH levels and reduce tumor size. Dopamine agonists, including cabergoline, are used in second-line treatment, but their efficacy is considerably lower than in prolactinomas.^11^

Pasireotide is a multi-receptor-targeted somatostatin transmembrane receptor (SSTR) ligand approved for patients with acromegaly or Cushing’s disease. Unlike the first-generation somatostatin analogs, lanreotide and octreotide, which preferentially bind SSTR2, pasireotide binds to multiple SSTRs with a high affinity for SSTR5 followed by SSTR2, SSTR3, and SSTR1. Pasireotide has shown effectiveness in normalizing GH and IGF-1 secretions, as well as reducing the pituitary mass both in medically naïve patients with active acromegaly after prior pituitary surgery and in treatment naïve *de novo* diagnosed patients.^12−14^ Preliminary reports and case studies suggest that it may also have utility in treatment-resistant macro-prolactinoma.^6,7,15,16^

Here we report on a rare difficult case of a patient with morbid obesity with an in-operable pituitary macroadenoma with hyperprolactinemia and acromegaly who failed to respond to octreotide therapy and was successfully managed with pasireotide.

## Case Presentation

A 48-year-old male was referred to our Endocrine clinic for obesity. He did not report any other symptoms. However, he reported that two years previously, he had been assessed in his home country for obesity, and routine workup indicated hormonal abnormalities. He had been diagnosed with a 3.5 × 3.5 cm pituitary macroadenoma on MRI. He received cabergoline 0.5 mg twice a week for 6 months to treat high prolactin levels. He subsequently discontinued this medication on his own and was lost to follow-up. His Epworth sleepiness score, to assess daytime sleepiness, was 6, which is normal.^17^ He was single and he never tried sexual intercourse or fathered a child. He was working as a laborer. He had no family history of pituitary diseases. On examination at our clinic, his blood pressure was 127/79 mmHg, his weight was 130 kg, and his BMI was 49.5 kg/m^2^. No remarkable findings were noted on general examination. Blood workup revealed hyperprolactinemia, with a level of 7,237 mIU/L (normal range: 85–323), acromegaly with an IGF-1 level of 450 ug/L (normal range: 88–210), and a positive GH suppression test. Secondary hypothyroidism [TSH: 2.52 mIU/L (normal range: 0.3–4.2), FT4: 9.7 pmol/L (normal range: 11.6–21.9)] and secondary hypogonadism [FSH: 1.2 IU/L (normal range: 1.5–12.4), LH: 1.1 IU/L (normal range: 1.7–8.6), testosterone: 0.14 nmol/L (normal range: 10.4–30.86)] were also observed, along with a confirmed normal adrenal axis on the short synacthen test. In addition, HbA1C was 6%, which was in the prediabetes stage (normal: < 5.7%). The baseline workup is summarized in Table 1.

**Figure 1.**
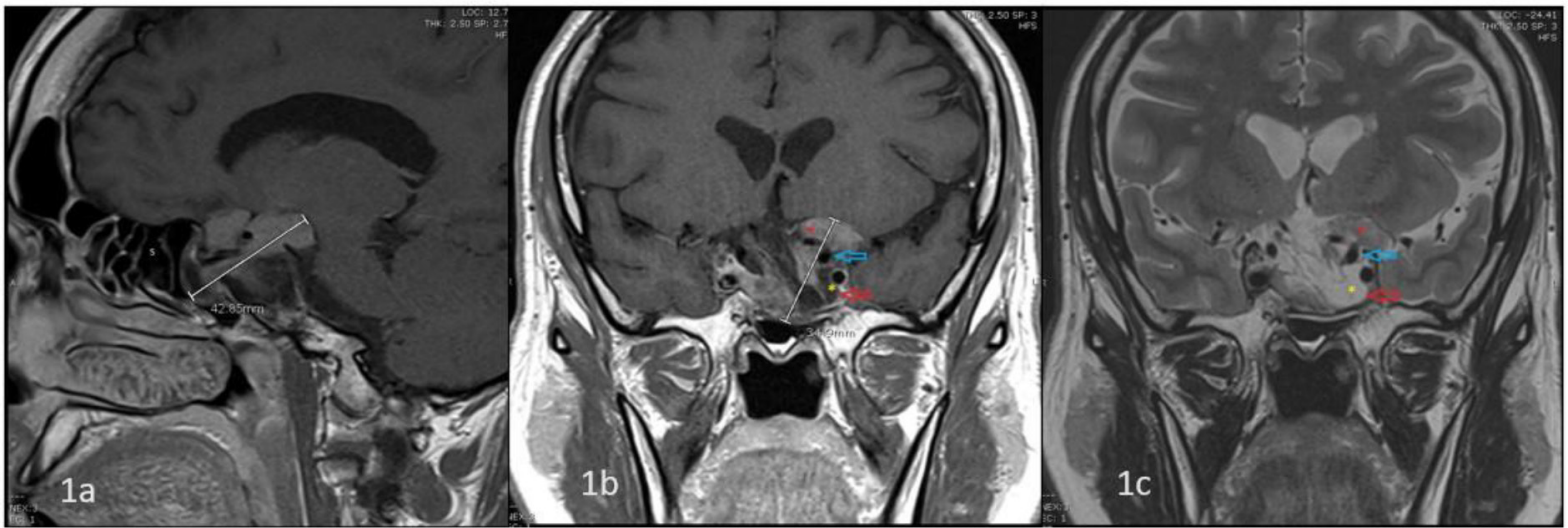
T1-weighted sagittal (a) and coronal (b) and T2-weighted coronal (c). Magnetic resonance imaging of the pituitary gland with intravenous contrast (pretreatment). The sella turcica is enlarged and bulging into the sphenoid sinus (S). There is a large sellar and suprasellar solid (red star) and cystic (yellow star) mass lesion. The solid component shows heterogeneous contrast enhancement. The cystic non-enhancing part extends into the left cavernous sinus (red arrow), and the solid suprasellar component encases the supraclinoid left ICA (blue arrow).

The patient underwent a pituitary MRI at our institution (Figure 1), which showed an irregular, mainly suprasellar T_1_ isointense, T_2_ dark, and homogenously enhancing pituitary macroadenoma (maximum axial diameter of about 29 mm). It encased parts of the left and right internal carotid arteries as well as ventral to the upper part of the basilar artery. The mass encroached on the left side of the optic chiasm and the prechiasmatic course of the left optic nerve. The pituitary stalk was displaced to the right side.

Perimetry assessment showed a constricted field mainly on the temporal side for the right eye and a constricted visual field for the left eye as well.

**Figure 2.**
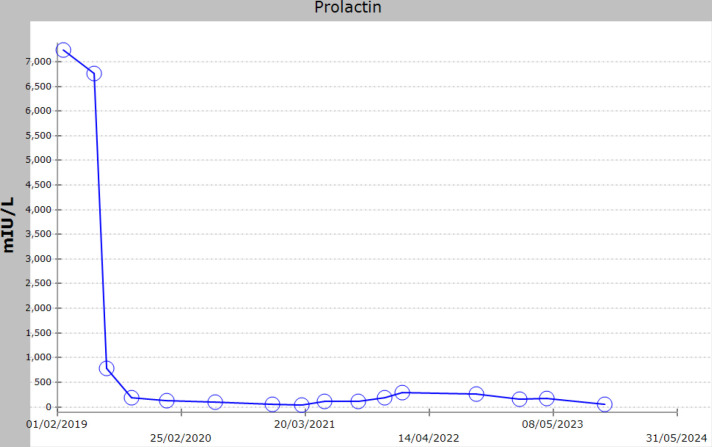
Prolactin levels over the course of the patient’s management.

**Figure 3.**
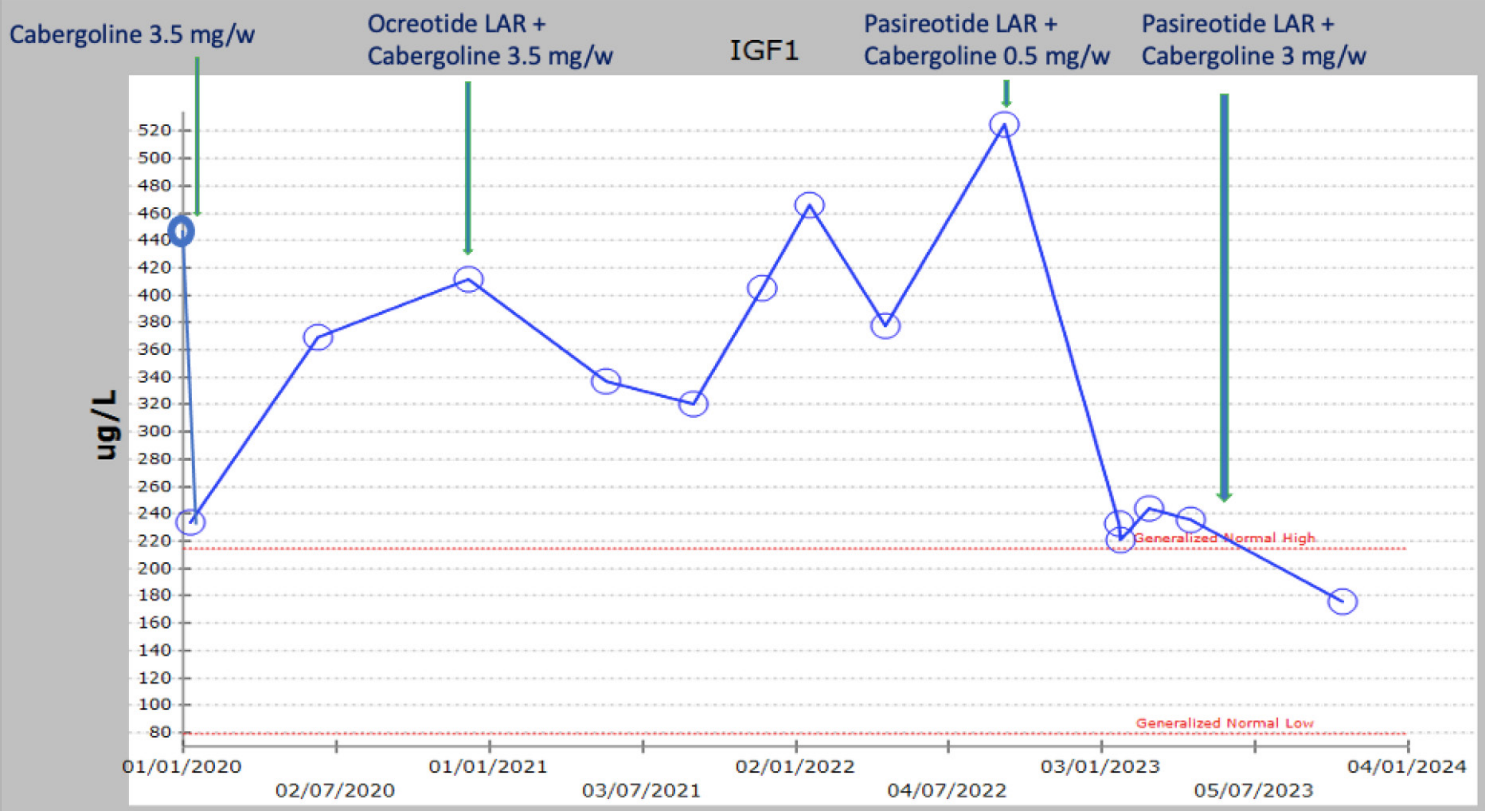
Changes in insulin-like growth factor 1 (IGF-1) levels over the course of the patient’s management.

Due to the extensive nature of the macroadenoma, complete surgical excision was deemed unfeasible. The patient was reluctant to undergo a debulking procedure. As the cost of octreotide was prohibitive for the patient, treatment with cabergoline was initiated, with the dose gradually increased to 0.5 mg daily. Additionally, he received replacement therapy with testosterone undecanoate intramuscular injections and levothyroxine. Prolactin levels normalized (Figure 2); however, IGF-1 did not improve (Figure 3). To address this, with the help of a charity, octreotide LAR was added at 20 mg every 4 weeks for 3 months, then escalated to 30 mg every 4 weeks for another 3 months, and further to 40 mg every 4 weeks for an additional 3 months; however, IGF-1 levels did not improve [IGF-1: 405 µg/L (normal: 79–214)]. Consequently, octreotide LAR was discontinued, and the patient was maintained on cabergoline monotherapy at the lowest effective dose to control hyperprolactinemia (0.5 mg/week). During this treatment course, regular examinations were conducted, including a pituitary MRI every 6–12 months, which showed no changes. Screening tests for acromegaly patients were unrevealing;^18^ echocardiography results were unremarkable, polysomnography found no obstructive sleep apnea, colonoscopy results were unremarkable, and no nodules were found on thyroid ultrasound. The patient was referred for radiotherapy and underwent two sessions but discontinued the treatment due to fear of side effects. He was referred to a dietitian to help with obesity management, where he lost 4 kg through dietary control. It is possible he could not reduce his weight more as he was a laborer, regularly eating commercially prepared meals.

**Figure 4.**
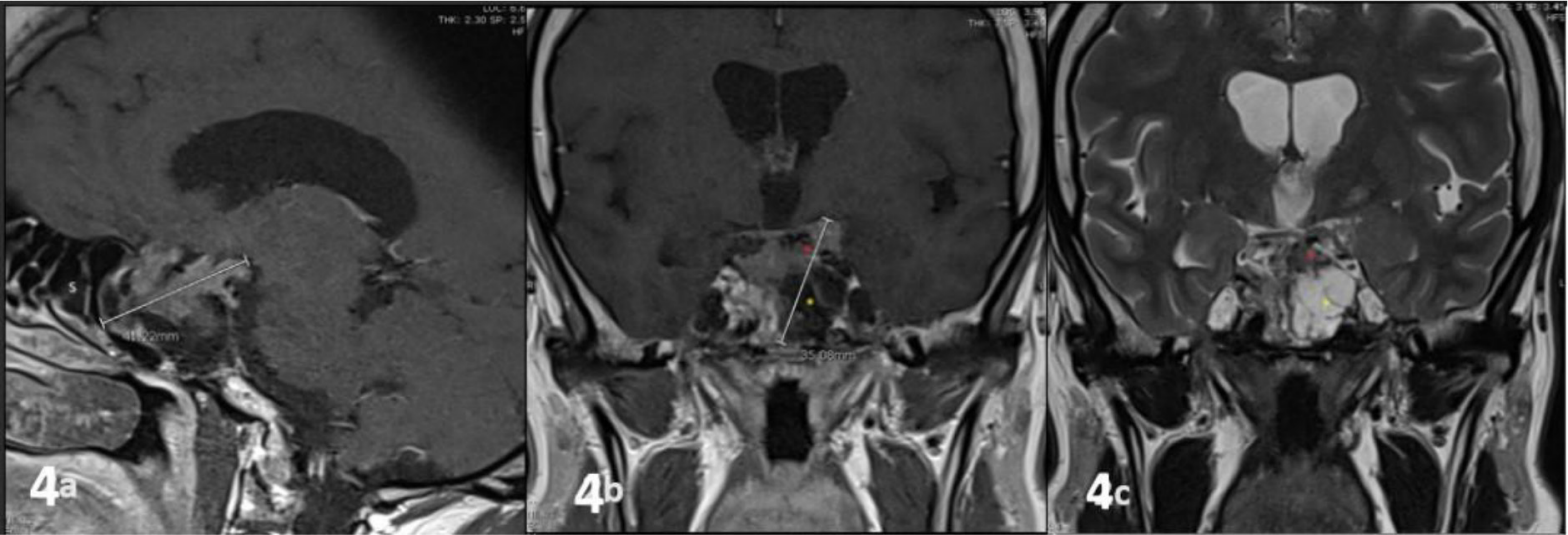
T1-weighted sagittal (a) and coronal (b), and T2-weighted coronal (c). Magnetic resonance imaging of the pituitary gland with intravenous contrast (post-treatment). There is no significant change in the size of the pituitary macroadenoma compared to the pretreatment pituitary MRI.

Recently, pasireotide became available at our institution, and the patient was initiated on pasireotide LAR 40 mg every 4 weeks in addition to the ongoing cabergoline 0.5 mg per week. Before starting pasireotide, the patient’s IGF-1 level was 525 µg/L (normal range: 79–214). It decreased to 221 ug/L after 3 months and remained stable after another 3 months. Thereafter, we increased cabergoline dose from 0.5 mg/week to 3 mg/week. Three months later, he reported that he felt generally better and more active. IGF-1 level became normal (176 µg/L). Growth hormone levels also decreased to 0.48 ug/L after 3 months and remained below 1 ug/L thereafter. Pituitary MRI after 6 months of pasireotide treatment showed no changes (Figure 4). However, after 6 weeks on pasireotide, the patient developed type 2 diabetes. Currently, his diabetes is well-controlled on metformin 1 g twice daily and sitagliptin 100 mg per day. Other common side effects of pasireotide are diarrhea, abdominal pain, and headache. The patient reported these side effects only in the 3–4 days after each injection. However, they did not stop him from continuing the medication. His current medication regimen includes pasireotide LAR 40 mg every 4 weeks, cabergoline 3 mg per week, levothyroxine 200 mcg per day, testosterone undecanoate 1 g every 12 weeks, metformin 1 g twice daily, and sitagliptin 100 mg per day.

## Discussion

Published case reports are mostly for patients with resistance acromegaly who responded to pasireotide.^19^ What distinguishes our case is that pasireotide has only recently been launched in our region, and thus there is limited clinical experience with its use by local endocrinologists and a corresponding limited awareness of its effectiveness in local populations. The case presented here highlights the successful management of a rare, difficult combination of acromegaly and hyperprolactinemia using pasireotide after the failure of both cabergoline monotherapy and cabergoline-octreotide combination therapy.

Acromegaly was diagnosed based on high IGF-1 and a positive GH suppression test.^18^ GH suppression test is recommended to confirm the diagnosis by finding a lack of suppression of GH below 1 µg/L after documented hyperglycemia during an oral 75 g glucose load.^18^ During the test, both glucose and GH are measured at baseline, then every 30 minutes for 2 hours after ingestion of 75 g glucose solution.^18^

The patient’s prolactin levels were effectively controlled throughout his treatment journey with low-dose cabergoline (0.5 mg/week), with no observable adverse events. However, persistently elevated IGF-1 levels were unresponsive to the first-generation somatostatin analog, octreotide LAR. The substitution of octreotide with pasireotide LAR led to a significant reduction in IGF-1 levels within 3 months of treatment.

While high prolactin levels in acromegaly are not uncommon,^3^ this particular case presented a significant challenge to manage due to an unfortunate convergence of multiple factors, including the inoperability of the tumor, the lack of response to first-line medical therapy, and the associated risks of radiotherapy for this pituitary adenoma. In this latter case, with the tumor encroaching on the left side of the optic chiasm and the prechiasmatic course of the left optic nerve, the patient’s vision was potentially at risk as a side effect of radiotherapy. Given this highly unusual convergence of limitations, the confirmation of an excellent clinical response to LAR pasireotide was a breakthrough finding.

The efficacy reported here with pasireotide compared with a non-response to octreotide is in line with the literature. In a randomized comparative study, pasireotide was shown to be superior to octreotide in uncontrolled acromegaly patients, with 15% of patients achieving control on pasireotide LAR 40 mg versus 0% on octreotide or lanreotide (*p* = 0.0006).^13^ In medically naïve acromegaly patients, significantly more pasireotide patients than octreotide patients achieved biochemical control (31% versus 19%; *p* = 0.007).^20^ This comparative potency over octreotide is likely to result from the binding profile of pasireotide to somatostatin receptors, with a higher affinity for SST3 and SST5 (5- and 40-times greater affinity than octreotide, respectively).^21^ However, pasireotide is associated with a greater incidence of hyperglycemia than octreotide,^22^ which was again confirmed in the present case, although the patient was effectively controlled with oral antidiabetic medications.

Although first-generation somatostatin analogs are widely used with considerable clinical efficacy in acromegaly, following unsuccessful surgery or in cases where surgery is not feasible, a substantial percentage of patients remain biochemically uncontrolled and subject to chronically elevated levels of GH and IGF-1.^23^ The reasons for poor response to first-generation somatostatin analogs are multifactorial, but likely include a low expression of SSTR2. Somatostatin receptor subtype 5 is also highly expressed in somatotropinomas, providing a rationale to explore the efficacy of pasireotide in these challenging patients.^23^

The clinical efficacy of pasireotide has indeed been well-documented in patients who have not responded to first-generation somatostatin analogs. Studies such as the PAOLA trial have shown that pasireotide can achieve normalization of IGF-1 levels in 25–26% at 6 months and in 33% at 18 months.^13,24^ Furthermore, in this cohort, 37% achieved GH < 1.0 µg/L and normal levels of IGF-I at some point during the study.^23^

The tumor size in our patient did not decrease after 6 months of treatment with pasireotide. However, it has been shown that patients who were uncontrolled on first-line medical therapy at maximum doses and for at least 6 months, then started to use pasireotide, got > 25% tumor shrinkage with pasireotide 40 mg and 60 mg in 19% and 11% of cases, respectively, versus only 2% who continued their first-line treatment.^23^ We plan to continue periodic (6–12 months) clinical, biochemical, and MRI pituitary follow-up to ensure biochemical control of his hormonal abnormalities and to ensure there is no increase in tumor size.

The long-term benefits of pasireotide in this patient population have also been confirmed in a variety of clinical and real-world settings, where 39–59% of patients who had previously failed on a first-generation somatostatin analog achieved normal IGF-1 levels on pasireotide beyond 6 months of treatment.^25−29^

This case aligns with recent personalized treatment recommendations that advocate for considering pasireotide in cases of first-generation somatostatin analog resistance when specific criteria are met.^30,31^ Pasireotide LAR as a relatively new and more expensive somatostatin analog is often used in non-responders to maximum tolerated doses of octreotide LAR or lanreotide. However, recent data suggest that it can be used as first-line therapy in patients with certain characteristics that predict resistance to first-generation somatostatin analogs.^31^ These criteria include T_2_ hyperintensity or hypointensity on MRI, low SSTR2 expression, high SSTR5 expression, substantial tumor volume, and specific genetic mutations.^19^ This underscores the importance of tailoring treatment approaches to individual patient profiles.

## Conclusion

This case of a pituitary macroadenoma with treatment-resistant acromegaly and hyperprolactinemia successfully managed with the introduction of pasireotide suggests that LAR pasireotide may have considerable utility in such patients where IGF-1 levels do not respond to standard treatment regimens, such as octreotide and/or cabergoline. Patients on pasireotide are at risk of developing hyperglycemia and must be monitored regularly; however, this can be managed with simple oral anti-diabetes medications.

## Funding

Medical writing and editorial assistance were provided by Guy Ramsay and funded by Recordati Rare Diseases.

## Conflict of Interest

No one of the authors received payment for this work. The fund provided by Recordati Rare Diseases was used only to cover medical writing and editorial assistance by Guy Ramsay.

## Figures and Tables

**Table 1.** Hormonal workup at presentation to our Endocrine clinic.

**Baseline hormonal workup:**		
**Laboratory variable**	**Result**	**Normal Range**
FSH (IU/L)	1.2	1.5–12.4
LH (IU/L)	1.1	1.7–8.6
Testosterone (nmol/L)	0.14	0.4–30.86
Prolactin (mIU/L)	7,237	85–323
TSH (mIU/L)	2.52	0.3–4.2
FT3 (pmol/L)	3.6	3.7–6.4
FT4 (pmol/L)	9.7	11.6–21.9
IGF-1 (ug/L)	450	88–210
ACTH (pg/ml)	117	7.2–63.3
Cortisol (nmol/L)	326	133–537
**Growth hormone (GH) suppression test:**		
**Time**	**GH (µg/L)**	**Glucose (mmol/L)**
Baseline	1.38	6.2
30-minute	1.43	10
60-minute	1.93	8.6
90-minute	1.79	7
120-minute	1.67	4.7
**Short Synacthen Test:**		
**Time**	**Cortisol (nmol/L)**	
Baseline	311	
30-minute	573	
60-minute	719	

FSH: Follicle Stimulating Hormone, LH: Luteinizing Hormone, TSH: Thyroid Stimulating Hormone, FT3: Free T3, FT4: Free T4, IGF-1: Insulin-like Growth Factor-1, ACTH: Adrenocorticotrophic Hormone.
